# Effect of body mass index and cholesterol‐rich apolipoprotein‐B‐containing lipoproteins on clinical outcome in NSCLC patients treated with immune checkpoint inhibitors‐based therapy: A retrospective analysis

**DOI:** 10.1002/cam4.7241

**Published:** 2024-05-31

**Authors:** Zixin Hu, Yumin Zheng, Jiabin Zheng, Yan Wang, Jiangquan Liao, Zhening Liu, Jia Li, Huijuan Cui

**Affiliations:** ^1^ Beijing Hospital of Traditional Chinese Medicine Capital Medical University Beijing China; ^2^ Department of Oncology China‐Japan Friendship Hospital Beijing China; ^3^ Beijing University of Chinese Medicine Beijing China; ^4^ Department of National Integrated Traditional and Western Medicine Centre for Cardiovascular Disease China‐Japan Friendship Hospital Beijing China

**Keywords:** body mass index, immune checkpoint inhibitor, low‐density lipoprotein cholesterol, non‐small cell lung cancer, remnant cholesterol

## Abstract

**Objectives:**

Obesity and hypercholesterolemia are linked to unfavor clinical outcomes. Recent studies declared the paradox that high body mass index (BMI) and serum cholesterol were independently connected to better clinical outcome of immune checkpoint inhibitors (ICIs) monotherapy in non‐small cell lung cancer (NSCLC). The aim of the study is to investigate the prognosis of BMI and serum cholesterol in ICIs‐based therapy.

**Methods:**

This is a retrospective study of 95 NSCLC patients treated with ICIs‐based therapy at the Department of Oncology and Lung Cancer Center of China‐Japan Friendship Hospital. Treatment efficacy was assessed using durable clinical benefit (DCB) versus nondurable benefit (NDB), best response (active vs. nonactive), and progression‐free survival (PFS). The prognostic value of BMI, LDL‐C, and RC was determined by multivariate regression analyses, while controlling for confounding factors including age, gender, diabetes status, smoking history, and statin usage. BMI was considered a confounding factor in the analysis when examining the impact of lipoproteins.

**Results:**

In our study, we found that in the whole group, BMI ≥25 kg/m^2^ was linked to a higher risk of poor therapeutic response (OR = 5.92, 95% CI 1.99–19.51, *p*.val = 0.002) and shorter progression‐free survival (HR = 3.00, 95% CI 1.59–5.68, *p*.val = 0.001). In addition, low levels of RC were associated with better therapeutic response (OR = 0.12, 95% CI 0.02–0.64, *p*.val = 0.019), while low levels of serum LDL‐C were found to predict longer PFS (HR = 0.40, 95% CI 0.19–0.82, *p*.val = 0.012). These associations were consistent in advanced NSCLC patients receiving ICIs and chemotherapy.

**Conclusions:**

Our study suggest that BMI ≥25 kg/m^2^ and elevated levels of apoB‐containing lipoproteins, including LDL‐C and RC, could potentially serve as useful prognostic markers for predicting poor treatment outcomes in advanced NSCLC patients treated with the combination of chemotherapy and ICIs.

## 1 INTRODUCTION

Non‐small cell lung cancer (NSCLC) accounts for approximately 85% of lung cancer, which stands as the primary cause of cancer‐related deaths.^1^ Immune checkpoint inhibitors (ICIs) that target the PD‐1/PD‐L1 axis have revolutionized the treatment of NSCLC without molecular targets. The standard first‐line treatment for NSCLC patients without sensitive mutations now involves a combination of chemotherapy and ICIs. This approach holds significance as chemotherapy can enhance the tumor's antigenicity to improve the efficacy, while reducing the occurrence of immune‐related adverse events (irAEs).^2^

Altered lipid metabolism is one of the most prominent metabolic changes observed in the tumor microenvironment (TME).^3^, ^4^ Recent research reported that patients with metabolic syndrome experience chronic inflammatory events and T cell exhaustion due to sustained upregulation of multiple inhibitory receptors and immunosuppressive cells.^5^, ^6^ More researchers are focusing on the impact of metabolic syndrome including hypercholesterolemia and obesity on the toxicity and efficacy of ICIs.

A notable “paradox” is the divergent effect of body mass index (BMI) on the clinical outcome of ICIs monotherapy and chemotherapy.^7^, ^8^ Specifically, high BMI was a protective factor for better outcomes of ICIs monotherapy in NSCLC patients, while a risk factor for those receiving chemotherapy. The prognostic significance of BMI in the context of combination therapy with chemotherapy and ICIs is currently a subject of controversy.^9^

Apolipoprotein B (apoB)‐containing lipoproteins, mostly including low‐density lipoprotein cholesterol (LDL‐C) and remnant cholesterol (RC), transport cholesterol in the bloodstream.^10^, ^11^ High levels of LDL‐C and apoB have been linked to an increased incidence of cancer and metastasis in most cancers.^12^ The relationship between cholesterol‐rich lipoproteins and ICIs therapeutic response remains unclear. On one hand, patients with high levels of LDL‐C, high‐density lipoprotein cholesterol (HDL‐C), and serum cholesterol have been shown to derive benefits from ICIs monotherapy regardless of PD‐L1 expression.^13^, ^14^ On the other hand, emerging evidence has indicated that statins, which lower serum cholesterol levels, may have addictive effect on the efficacy of ICIs monotherapy.^15^

We conducted a retrospective case–control analysis at the Department of Oncology and Lung Cancer Center of China‐Japan Friendship Hospital to investigate the impact of BMI and apoB‐containing lipoproteins on the clinical outcome of chemotherapy and ICIs combination regimens in NSCLC patients. The study aimed to declare the prognostic effect of metabolic biomarkers (BMI and serum lipoproteins) on the clinical outcomes of chemoimmunotherapy in NSCLC.

## 2 MATERIALS AND METHODS

### 2.1 Patient cohorts

In the present study, we conducted a retrospective analysis of NSCLC patients without driver genes who received ICIs‐based therapy for the first time, irrespective of treatment line, between January 2019 and December 2021 at the Department of Oncology and Lung Cancer Center of China‐Japan Friendship Hospital. The pathological diagnosis of NSCLC was categorized based on the 2015 World Health Organization Classification of Tumors, while the disease stage was evaluated using version eight of the tumor, node, metastasis system (TNM) staging classification.

The inclusion criteria for enrolled patients were as follows: 1. Histologically‐ and radiologically‐confirmed Stage III‐IV NSCLC; 2. Administration of chemoimmunotherapy or ICIs for the first time, regardless of treatment line and PD‐L1 status. The chemotherapy and immunotherapy regimens were formulated based on the guidelines of the National Comprehensive Cancer Network (NCCN); 3. The Eastern Cooperative Oncology Group Performance Status (ECOG‐PS) score for all patients ranged from 0 to 1. The exclusion criteria were as follows: 1. inability to undergo radiographic evaluation; 2. receiving radiotherapy or cryoablation therapy for lung or mediastinal lesions; 3. stopping the use of ICIs prior to the first evaluation of treatment efficacy.

Radiographic tumor responses were evaluated using the Response Evaluation Criteria in Solid Tumors (RECIST) version 1.1^16^ and were independently evaluated by two radiologists. Progression‐free survival (PFS) was calculated from the initial of ICIs‐based therapy to disease progression. Patients without documented disease progression were censored on the date of last imaging follow‐up (May 1, 2022). Stable disease (SD) was classified as SD‐a when there was not enough shrinkage to qualify for a partial response (PR), and as SD‐b when there was not enough increase to qualify for progressive disease (PD). If the target lesion did not exhibit any radiological changes, the response was considered SD.

The clinical response to treatment was assessed using two methods. Firstly, durable clinical benefit (DCB) was defined as a partial response (PR) or stable disease (SD) that lasted for more than 6 months, while nondurable benefit (NDB) was considered as progressive disease (PD) or SD lasting less than 6 months.^17^ In addition, the best response to treatment was evaluated and categorized as active or nonactive response. Patients who achieved a PR or SD‐a were classified as having an active response. Those who did not achieve PR or SD‐a were categorized as having a nonactive response. Immune‐related adverse events (irAEs) were graded according to the US National Cancer Institute Common Toxicity Criteria for Adverse Events (NCI‐CTCAE; version 5.0).

We followed all ethical guidelines for working with human participants and obtained informed consent from the participants. Additionally, this study was approved by the institutional review board at China‐Japan Friendship Hospital (2022‐KY‐051).

### 2.2 BMI evaluation

Baseline demographic and clinical data were collected from the medical records of patients at their first administration of ICIs or during ICIs treatment. The pretreatment height and body weight of each patient were recorded to calculate BMI, which was classified according to the World Health Organization (WHO) categories: underweight, BMI < 18.5 kg/m^2^; normal, 18.5 kg/m^2^ ≤ BMI ≤24.9 kg/m^2^; overweight, 25 kg/m^2^ ≤ BMI ≤29.9 kg/m^2^; obesity, BMI ≥30 kg/m^2^. Patients were categorized based on their BMI as either BMI ≥25 kg/m^2^ or BMI < 25 kg/m^2^ for the further analysis.

### 2.3 ApoB‐containing lipoproteins evaluation

Serum levels of total cholesterol (TC) (mmol/L), total triglycerides (TG) (mmol/ L), HDL‐C (mmol/L), LDL‐C (mmol/L), apoA1 (g/L), and apoB (g/L) were measured. TC, TG, LDL‐C, and HDL‐C were measured using a Beckman Coulter kit, while apolipoproteins were measured using a Nittbo Medical Co.LTD kit. RC content of non‐LDL‐C and non‐HDL‐C was calculated as TC minus LDL‐C and HDL‐C. Patients were divided into two groups based on the threshold levels of serum LDL‐C and RC. The thresholds for LDL‐C and RC were determined using the “surv‐cutpoint” function in the survminer R package since no information about the cutoff points of these markers in NSCLC was available.

### 2.4 Statistical analysis

The statistical analysis was performed using R software (version 4, 4.0.4), and the sample size was calculated based on the expected OR = 2, *α* = 0.05 and *β* = 0.10 for the primary endpoint (NDB/DCB). Baseline characteristics were presented as mean and standard deviation (SD) or median and interquartile range (IQR) for continuous variables, and frequencies and percentages for categorical variables. The comparison between groups was performed using *t*‐test or Mann Whitney *U* test, while the chi‐square test was used to estimate the distribution of groups. Missing values were handled using listwise deletion, which is the default method in R.

Multivariate logistic model were employed to examine the effect of BMI, LDL‐C, and RC on therapeutic result, including DCB/NDB and the best response. PFS was compared between groups with BMIs of 25 kg/m^2^ or below, DCB and NDB groups, and active and nonactive response groups. Kaplan–Meier (KM) method and log‐rank test for trends were used to evaluate and compare PFS. Cox proportional hazards models were used to analyze the association between variables and PFS. The multivariate models were used to adjust the confounders including age, gender (male or female), diabetes history (yes/no), smoking history (yes/no), and statin usage (yes/no). When analyzing LDL‐C and RC, BMI was considered as a confounding factor to include in the model. Both continuous and categorical versions of lipoproteins were tried to be included in models to obtain the better models for lipoproteins. Subgroup analyses were used in the stage IIIb‐IV cohort, the chemo+ICIs cohort, and the first‐line ICIs‐based therapy cohort.

Receiver operating characteristic (ROC) curves were used to assess the discriminative ability of markers by the pROC package, and time‐dependent ROC curves at 3 years of the Cox model were plotted using the timeROC R package. The calibration analysis was carried out by calibrate function embedding in the rms R package. The efficiency of models was evaluated by comparing decision curve analysis (DCA) curves of models. All *p*‐values were two‐sided, with confidence intervals (CIs) set at 95% and significance defined as <0.05.

## 3 RESULTS

### 3.1 Clinical characteristics of enrolled patients

A total of 98 patients with NSCLC who received the combination of chemotherapy and ICIs (chemo+ICIs) or ICIs single regimen were enrolled in China‐Japan Friendship Hospital during January 2019 to December 2021. Three patients did not complete the first evaluation for their initial treatment, and therefore, only 95 patients were considered for analysis. The baseline clinical features of these patients are summarized in Table 1 and Table S1. Of the 95 patients, 82 (86.3%) were treated with chemo+ICIs combination regimens, while 13 received ICI monotherapy. Most patients (90/95, 94.7%) used anti‐PD1 therapy. The majority of patients (68/95, 71.6%) received chemo+ICIs/ICIs as their first‐line treatment.

**TABLE 1:** Baseline clinical features of the 95 patients included in the analysis.

Variable	Median (95% CI)/Frequency	N	Variable	Frequency	N
Age	64.0 [58.0;69.0]	95	Responsiveness		74
Gender			DCB	43 (58.1%)	
Female	14 (14.7%)	95	NDB	31 (41.9%)	
Male	81 (85.3%)		Best response		85
Stage		95	Active	58 (68.2%)	
IIIA	9 (9.47%)		Nonactive	27 (31.8%)	
IIIB‐IV	86 (90.5%)		AE		95
Histology		95	N	57 (60.0%)	
LUAD	55 (57.9%)		Y	38 (40.0%)	
LUSC	33 (34.7%)		Smoking history		93
NSCLC	6 (6.32%)		N	24 (25.8%)	
NSCLC+SCLC	1 (1.05%)		Y	69 (74.2%)	
Treatment plan		95	DM		93
Chemo+ICIs	82 (86.3%)		N	76 (81.7%)	
ICIs	13 (13.7%)		Y	17 (18.3%)	
ICIs		95	Statins		95
Anti‐PD1	90 (94.7%)		N	65 (68.4%)	
Anti‐PDL1	2 (2.1%)		Y	30 (31.6%)	
Multi	3 (3.2%)		BMI		95
Line		95	Normal	56 (58.9%)	
1	68 (71.6%)		Overweight	34 (35.8%)	
2+	27 (28.4%)		Obesity	5 (5.26%)	

Abbreviations: AE, adverse event; DCB, durable clinical benefit; LUAD, lung adenocarcinoma; LUSC, lung squamous cell carcinoma; NDB, nondurable benefit; NSCLC, non‐small cell lung carcinoma.

The median age of the 95 patients was 64 years (IQR 58–69), and the majority were male (84/95, 85.3%). Among them, 55 patients (57.9%) were diagnosed as lung adenocarcinoma (LUAD), 33 patients (34.7%) was lung squamous cell carcinoma (LUSC), while others had large cell carcinomas or mixed NSCLC. There were 86 patients (90.5%) with stage IIIb‐IV tumors, and nine patients (9.5%) with stage IIIA tumors.

There were no underweight patients in the study population. The overweight and obese individuals were grouped together as BMI ≥25 kg/m^2^. Of the 95 patients, 56 (58.9%) were BMI < 25 kg/m^2^, while 39 (41.1%) were BMI ≥25 kg/m^2^. No significant difference was observed between the BMI < 25 kg/m^2^ group and BMI ≥25 kg/m^2^ group in terms of baseline clinical characteristics, serum lipids, lipoproteins, and apolipoproteins, as shown in Table S2 and S3.

For this investigation, the average follow‐up duration was 9.6 months. Out of the 95 patients, 74 were followed up for a duration of at least 6 months and had the primary endpoint (DCB or NDB). Nine patients were in follow‐up; nine patients had their ICIs stopped due to severe side effects; two patients declined treatment; and two patients did not receive an examination on time. For 85 patients, response information(active or nonactive) was available. Due to serious adverse events, four patients had their ICIs terminated, four patients were in follow‐up, and two patients chose not to receive treatment. The clinical demographics were generally well‐balanced between the NDB and DCB groups (Table S4) and the active and nonactive response groups (Table S6).

As of May 1st, 2022, irAEs were recorded for all 95 patients, with 38 (40.0%) patients experiencing irAEs and nine patients stopping ICIs due to irAEs. No significant difference in body mass index (BMI) was observed between patients who experienced irAEs and those who did not. There was no statistical difference in the prevalence of serum lipids, lipoproteins, and apolipoproteins between patients who developed irAEs and those who did not.

### 3.2 Association between BMI and therapeutic response in ICIs‐based therapy

**FIGURE 1:**
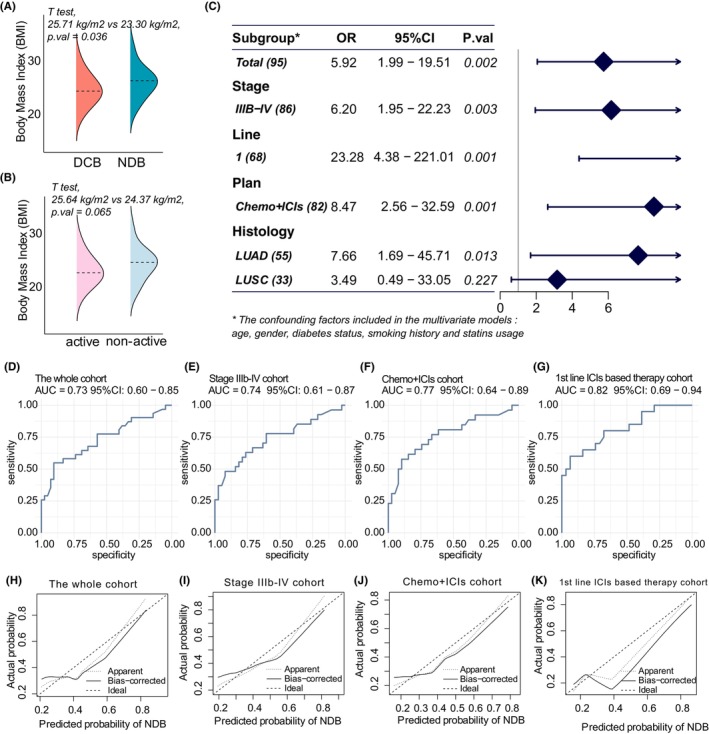
Associations between body mass index (BMI) and therapeutic response to immune checkpoint inhibitors (ICIs)‐based therapy in non‐small cell lung cancer patients. (A) Comparison of the BMI prevalence between patients with durable clinical benefit (DCB) and nondurable benefit (NDB). (B) Comparison of the BMI prevalence according to the best response to the ICIs‐based therapy. (C) Subgroup analysis of the multivariate model to predict nondurable benefit (NDB). BMI ≥25 kg/m^2^ identified as a risk factor for NDB, with the odds ratio (OR) indicating the increased risk associated with BMI ≥25 kg/m^2^. (D–G) ROC curves of the multivariate model containing BMI ≥25 kg/m^2^ in predicting response to ICIs‐based therapy in the whole group and subgroups. (H–K) Calibration curves of the model containing BMI ≥25 kg/m^2^ in predicting therapeutic response in the whole group and subgroups.

Patients with NDB were more likely to have a higher BMI compared to those with DCB (*t*‐test, 25.71 kg/m^2^ vs. 23.30 kg/m^2^, *p*.val = 0.036, Figure 1A). Patients with nonactive response tended to have higher BMI than those with active response, despite not statistically significant (*t*‐test, 25.64 kg/m^2^ vs. 24.37 kg/m^2^, *p*.val = 0.062, Figure 1B).

Individuals with BMI greater than 25 kg/m^2^ were found to have a higher likelihood of NDB and nonactive response to ICIs‐based therapy after adjusting for confounders such as age, gender, diabetes status, smoking history, and statin usage. The OR for deriving NDB was 5.92 (95% CI 1.99–19.51, *p*.val = 0.002, Figure 1C), and the OR for deriving nonactive response was 4.88 (95% CI 1.76–14.59, *p*.val = 0.003, Figure S1A). In the subgroup analysis, BMI ≥25 kg/m^2^ was a risk factor for NDB in stage IIIb‐IV malignancies, chemo+ICIs combination group, first‐line ICIs treatment plan, and LUAD subgroups (Figure 1C, Figure S1A). BMI ≥25 kg/m^2^ was associated with an increased risk of NDB and nonactive response in patients with advanced NSCLC undergoing combination chemotherapy and immune checkpoint inhibitors (OR = 6.73, 95% CI 1.99–26.08, *p*.val = 0.003; OR = 4.36, 95% CI 1.37–15.12, *p*.val = 0.015).

We used the model diagnosis technique in four groups: the entire group, the stage IIIb‐IV group, the chemo+ICIs combination group, and the first‐line treatment group, in order to assess the multivariate model's efficacy. For DCB prediction, the area under the ROC curve (AUC) was 0.73, 0.74, 0.77, and 0.82, with corresponding 95% confidence intervals of 0.60–0.85, 0.61–0.87, 0.64–0.89, and 0.69–0.94 (Figure 1D–G). The AUC for predicting an active response to treatment was 0.70, 0.71, 0.72, and 0.72, with corresponding 95% confidence intervals of 0.57–0.84, 0.57–0.85, 0.57–0.87, and 0.53–0.90 (Figure S1B–E). Nevertheless, the calibration curves demonstrated that the multivariate models with BMI category tended to underestimate the likelihood of NDB and nonactive response (Figure 1H–K, Figure S1F–I).

### 3.3 Association between BMI and PFS in ICIs‐based treatment

**FIGURE 2:**
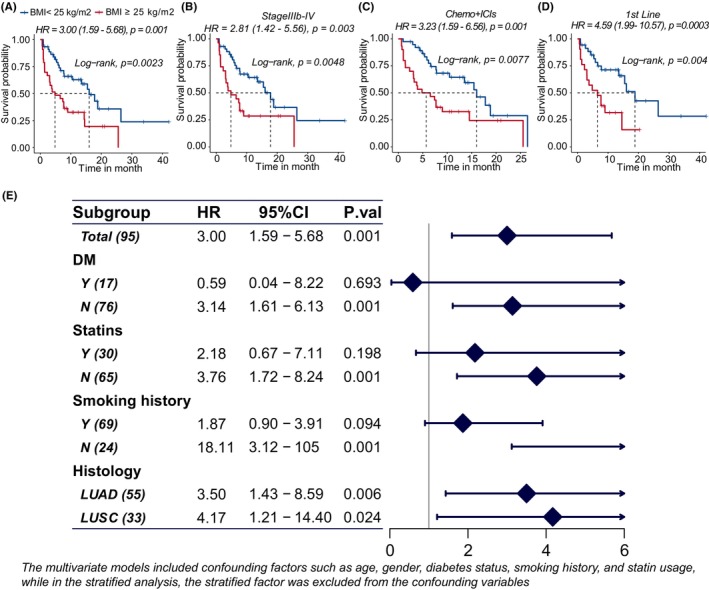
Association between BMI and progression‐free survival (PFS) in NSCLC patients undergoing ICIs‐based therapy. (A–D) Kaplan–Meier curves comparing patients with BMI ≥25 kg/m^2^ and BMI <25 kg/m^2^ in the entire cohort and subgroups. Patients with BMI ≥25 kg/m^2^ demonstrated consistently shorter progression‐free survival (PFS) across all cohorts. (E) Stratified analysis of the multivariate Cox model incorporating BMI to predict PFS in the entire cohort. The hazard ratio (HR) represents the increased risk associated with patients with BMI ≥25 kg/m^2^, indicating a higher likelihood of shorter PFS.

Patients with normal weight had a longer median PFS as compared to those with BMI ≥25 kg/m^2^ (Log‐rank test, 16.03 months vs. 4.83 months, *p*.val = 0.002, Figure 2A). Moreover, patients with normal weight had longer PFS in the stage IIIb‐IV subgroup, chemo+ICIs subgroup, and the 1st line subgroup (*p*.val = 0.005, 0.008, 0.004, respectively, Figure 2B–D). The advanced NSCLC patients with a BMI ≥25 kg/m^2^ defined a subgroup who derived less benefit from the combination of chemotherapy and ICIs with inferior PFS after adjusting for confounding factors (HR = 2.68, 95% CI 1.28–5.58, *p*.val = 0.009).

Model diagnosis of the multivariate model in predicting PFS were applied in the total group and the subgroups, respectively. The C‐index of the multivariate Cox model was 0.68, 0.68, 0.68, and 0.71 (95% CI 0.60–0.76, 0.60–0.77, 0.60–0.77, 0.63–0.80). The 3‐year AUC was 0.76, 0.72, 0.85, and 0.81 (Figure S2A–D), and the calibration curve was shown in Figure S2E–H. We further applied a stratified analysis according to the diabetes history, statin usage, smoking history, and histology. BMI ≥25 kg/m^2^ was a risk factor for inferior PFS especially in the patients without diabetes, nonstatin users and nonsmokers (Figure 2E).

### 3.4 Association between remnant cholesterol levels and response to ICIs‐based therapy

Compared to DCB patients, NDB patients had higher prevalence of serum cholesterol, RC, and LDL‐C, but the difference was only statistically significant for RC (*t*‐test, 0.64 mmol/L vs. 0.49 mmol/L, *p*.val = 0.042, Table S5). No significant differences were observed in circulating lipids, lipoproteins, and apolipoproteins between the active and nonactive groups in the total NSCLC cohort (Table S7).

Based on the PFS of the treatment, the total group was divided into an RC high group and an RC low group according to their serum RC levels. Similarly, the total cohort was also grouped by LDL‐C level and apoB level. The cut points for RC and LDL‐C were selected as 0.82 mmol/L and 2.58 mmol/L, respectively.

Low RC is linked with the clinical benefit of ICIs‐based therapy in the overall group, the stage IIIb‐IV subgroup, the chemo+ICIs combination subgroup, and the first‐line treatment subgroup. Low RC (both continuous and categorical) was a protective factor for better clinical outcome (DCB and active response), as demonstrated in Table 2. The advanced NSCLC patients were more likely to derive clinical benefits from the combination regimen of chemotherapy and ICIs (HR = 0.08, 95% CI 0.01–0.51, *p*.val = 0.012).

**TABLE 2:** The effect of remnant cholesterol (RC) in continuous and category form on the responsiveness to immune checkpoint inhibitors (ICIs)‐based therapy.

Variable^a^	ICIs response (NDB)			Nonactive response to ICIs		
	OR	95% CI	*p* Value	OR	95% CI	*p* Value
The whole cohort						
RC	10.55	1.32–106.34	0.033	4.77	0.66–39.42	0.128
Low RC	0.12	0.02–0.64	0.019	0.23	0.05–1.05	0.062
Stage IIIb‐IV						
RC	11.32	1.21–139.06	0.041	4.42	0.49–46.11	0.191
Low RC	0.12	0.02–0.69	0.023	0.24	0.04–1.25	0.093
Chemo + ICIs						
RC	22.39	2.26–327.38	0.013	8.29	1.02–83.22	0.055
Low RC	0.07	0.01–0.44	0.008	0.17	0.03–0.79	0.029
1st line						
RC	27.46	1.18–1108.81	0.052	5.76	0.43–100.53	0.199
Low RC	0.04	0–0.44	0.02	0.20	0.03–1.31	0.096

Abbreviations: 1st line, ICIs‐based therapy intervention in the first line; Chemo+ICIs, chemotherapy plus ICIs combination therapy; NDB, nondurable benefit.

^a^ The odds ratio (OR) was estimated using a multivariate logistic model with RC (continuous or categorical). The following confounding factors were included in the multivariate models: age, gender, diabetes status, BMI category, smoking history, and statins use. The model was run in both the entire group and subgroups.

We performed model diagnosis to evaluate the ability of RC in predicting the response to ICIs. The AUC of the multivariate model containing RC level (continuous) or low RC for predicting DCB in the whole cohort, stage IIIb‐IV subgroup, chemo+ICIs subgroup, and 1st line subgroup were shown in Figure S3A–H. The calibration curves of the models were shown in Figure S3I–P.

### 3.5 Association between LDL‐C levels and PFS in ICIs‐based treatment

**FIGURE 3:**
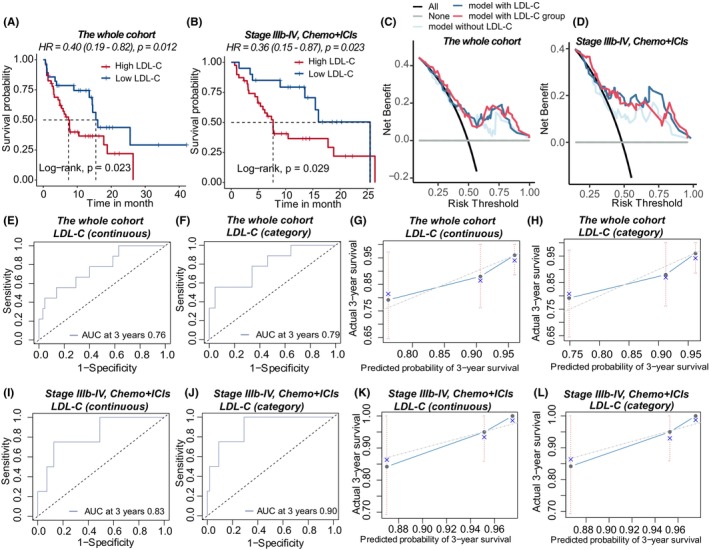
Association between low‐density lipoproteins cholesterol (LDL‐C) and progression‐free survival (PFS) in NSCLC patients undergoing ICIs‐based therapy. (A, B) Kaplan–Meier curves showing the PFS differences between patients with low and high LDL‐C in the entire cohort and in the subset of patients with advanced non‐small cell lung cancer (NSCLC) receiving chemotherapy and ICIs. (C, D) Decision curve study of models with and without LDL‐C in predicting outcomes in the total cohort and the subgroup with advanced NSCLC receiving chemotherapy and ICIs. (E, F) ROC curves of the models containing LDL‐C (continuous and category) in the whole cohort. (G, H) Calibration curves of the models containing LDL‐C (continuous and category) at 3 years in the whole cohort. (I, J) ROC curves of the models containing LDL‐C (continuous and category) in the advanced NSCLC treated by ICIs and chemotherapy subgroup. (K, L) Calibration curves of the model containing LDL‐C (continuous and category) at 3 year in the advanced NSCLC treated by ICIs and chemotherapy subgroup.

Patients with elevated LDL‐C had a shorter PFS (Log‐rank test, median PFS: 15.5 months vs. 7.4 months, *p*.val = 0.006, Figure 3A). According to the multivariate Cox model (Table 3), low LDL‐C was determined to be a protective factor for longer PFS. Patients with advanced NSCLC who had low LDL‐C were more likely to respond to ICIs plus chemotherapy (HR = 0.36, 95% CI 0.15–0.87, *p*.val = 0.023, Figure 3B).

**TABLE 3:** The effect of low‐density lipoprotein cholesterol (LDL‐C) in continuous and category form on progression‐free survival (PFS) in patients receiving immune checkpoint inhibitors (ICIs)‐based treatment.

Variable^a^	HR	95% CI	*p* Value
The whole cohort			
LDL‐C	1.50	1.01–2.22	0.043
Low LDL‐C	0.40	0.19–0.82	0.012
Stage IIIb‐IV			
LDL‐C	1.52	0.99–2.33	0.057
Low LDL‐C	0.40	0.19–0.88	0.022
Chemo + ICIs			
LDL‐C	1.60	1.05–2.44	0.028
Low LDL‐C	0.38	0.17–0.86	0.020
1st line			
LDL‐C	1.27	0.72–2.24	0.407
Low LDL‐C	0.35	0.14–0.87	0.023
Stage IIIb‐IV & Chemo + ICIs			
LDL‐C	1.70	1.07–2.7	0.025
Low LDL‐C	0.36	0.15–0.87	0.023

Abbreviations: 1st line, ICIs‐based therapy intervention in the first line; Chemo+ICIs, chemotherapy plus ICIs combination therapy; HR, hazard ratio.

^a^ The hazard ratio (HR) was estimated using the multivariate Cox models with LDL‐C (continuous or categorical), adjusting for the confounders including age, gender, smoking history, diabetes status, statin usage, and BMI.

The DCA curves demonstrated that adding LDL‐C to the model gave additional utility when compared to the model without LDL‐C in both the total group and advanced NSCLC patients treated with chemo+ICIs (Figure 3C,D). The C‐index of the model containing LDL‐C (continuous) or low LDL‐C (categorical) was 0.70 and 0.70 (95% CI 0.62–0.77, 0.63–0.79), respectively. The model ROC curves and calibration curves were shown in Figure 3E–H. The C‐index of the models containing LDL‐C in advanced NSCLC patients treated with chemo+ICIs was 0.68 and 0.70 (95% CI 0.59–0.78, 0.60–0.79). Model diagnostics including ROC curves and calibration curves were shown in Figure 3I–L.

## 4 DISCUSSION

In this study, we examined the impact of BMI and serum apoB‐containing lipoproteins (remnant cholesterol and LDL‐C) on the efficacy of ICIs‐based therapy in treating NSCLC patients who were receiving ICIs for the first time, regardless of their PD‐L1 status. Our study showed that a BMI ≥25 kg/m^2^ was linked to unfavorable clinical outcomes in advanced NSCLC patients who were treated with a combination of chemotherapy and ICIs. Furthermore, elevated levels of apoB‐containing lipoproteins, including LDL‐C and remnant cholesterol, were identified as risk factors for poor response to the combination treatment in advanced NSCLC. These findings indicate the potential usefulness of noninvasive biomarkers for predicting the effectiveness of ICIs‐based therapies, particularly the combination of ICIs and chemotherapy regimens.

The increasing prevalence of obesity and hypercholesterolemia is linked to an elevated risk of cancer‐related mortality. Excess body weight and hypercholesterolemia are associated with poor clinical outcomes in cancer treatment due to the chronic inflammatory state‐induced T cell exhaustion. However, recent studies have emphasized the “paradox”, whereby elevated BMI and hypercholesterolemia were both independently associated with improved outcomes in cases treated by ICIs monotherapy in multiple cancers.

According to studies by Cortellini et al. and Hisao Imai et al., high BMI was found to be a protective factor for better ICIs response in first‐line treatment with high PD‐L1 expression, as well as second‐line treatment regardless of PD‐L1 expression in NSCLC.^7^, ^8^, ^18^ In the Asian study, the cutoff for BMI was lower than that of the study in Western countries. However, despite the difference in cutoff value, the protective effect of high BMI on the clinical response to ICIs monotherapy remained consistent across both studies. There is growing evidence to suggest that the impact of BMI on ICIs response is more likely related to immune‐modulatory effects rather than nutritional factors, despite some suggestions that patients with cancer cachexia syndrome may have reduced ICIs response.^19^, ^20^ Further researches about the impact of BMI on the clinical outcome of ICIs monotherapy are necessary due to the limited evidence available.^21^

In contrast to ICI monotherapy, high BMI was found to be a risk factor for the treatment outcome of chemotherapy.^7^ Due to the joint effects of chemotherapy and ICIs, the impact of BMI on the combination regimen remains unclear.^9^ In our study, we found that high BMI was a risk factor for the clinical outcome of combination treatment. The divergent effects of high BMI on ICIs monotherapy and chemotherapy reported in previous studies suggest distinct immune regulatory roles between chemotherapy and immunotherapy, which may help explain the results observed in our study.^8^ Although our study did not find a relationship between BMI and irAEs, previous research has shown that higher pretreatment BMI is linked to a higher incidence of irAEs of any grade.^22^, ^23^ Thus, more research is required to figure out the association between BMI and chemoimmunotherapy.

In previous studies, it has been demonstrated that elevated serum cholesterol levels are linked to the efficacy of monotherapy with immune checkpoint inhibitors (ICIs) in advanced cancer patients. This is believed to be attributed to the presence of low‐grade inflammation state in these patients.^24^ High levels of cholesterol in the tumor microenvironment have been shown to induce exhaustion of CD8+ T‐cells, leading to an upregulation of immune checkpoint expression.^25^ Apolipoprotein B‐containing lipoproteins, such as LDL‐C and remnant lipoproteins like small VLDL, IDL, and chylomicron remnants, play a critical role in transporting cholesterol.^11^, ^25^ Studies have demonstrated that elevated levels of LDL‐C are positively correlated with a response to ICIs monotherapy.^13^, ^14^

There is ongoing debate on the predictive significance of cholesterol in the reaction to ICI monotherapy. Cancer cells with soft, cholesterol‐rich plasma membranes can obstruct T‐cells from destroying cancer cells, according to a recent study. Nevertheless, it has been demonstrated that depleting cholesterol increases T‐cell cytotoxicity and boosts the efficacy of adoptive T‐cell treatment.^26^ Statins target the rate‐limiting enzyme in cholesterol biosynthesis, leading to a reduction in serum levels of apoB‐containing lipoproteins and a lowering of cholesterol. The concurrent use of statins during ICI treatment has been shown to have a positive impact on treatment outcomes. One possible explanation for this is that statins can alter the tumor microenvironment by reducing the recruitment of immune cells that are known to promote tumorigenesis.^27^ Research is currently ongoing to determine the function of serum cholesterol in the response to chemotherapy and ICIs. As the primary receptor for apoB‐containing lipoproteins across various cell types, including T cells and NK cells, the low‐density lipoprotein receptor (LDLR) has the potential to function as an immune checkpoint akin to molecules like Lymphocyte activation gene‐3 (LAG‐3), T cell immunoglobulin and mucin‐domain containing protein‐3 (TIM‐3), and HLA‐G.^26^, ^28^

Given the increasing popularity of combining adjunctive medications like vitamin D and microbiota to enhance the efficacy of ICIs,^29^, ^30^ this study offers insights into the potential feasibility of combining statins, ω‐3 fatty acids, and proprotein convertase subtilisin/kexin type 9 (PCSK9) inhibitors.^31^, ^32^ These medications have the capacity to lower serum levels of apoB‐containing lipoproteins, presenting a promising approach to optimize the therapeutic effects of ICIs.

The current study has several limitations, including the single‐center cohort and the small sample size. Despite efforts to manage missing data, its presence may introduce unintended bias or unpredictability. For logistic and Cox model development, we employed listwise elimination for its simplicity and ability to minimize inaccurate inferences regarding missing data. However, this method carries a risk of bias if the missing data lack randomness, potentially reducing statistical power by decreasing the sample size. Therefore, we conducted discrimination, calibration, and efficiency analyses to assess the model's performance.

Secondly, we recognized that our cohort was heterogeneous and hence performed subgroup analysis. The predictive roles of BMI and apoB‐containing lipoproteins were consistent across the entire cohort as well as the subgroup cohorts (stage IIIb‐IV, chemo+ICIs combination, and first‐line therapy). We also ran model diagnosis analyses in the entire cohort and each subgroup. The prognostic roles of BMI and apoB‐containing lipoproteins were consistent across the whole cohort and the subgroup cohorts.

Thirdly, there were only five obese patients and no underweight patients, therefore the impact of obesity and underweight on the effectiveness of ICIs‐based therapy was not assessed. Furthermore, we only included patients with an ECOG‐PS of 0–1, as cancer cachexia is another factor that can skew the results. The ECOG‐PS score of each patient in our dataset ranged from 0 to 1, therefore results for individuals with worse baseline conditions were not available.

Moreover, there was no discernible impact of BMI or lipoproteins containing apoB on irAEs. There was event effect bias in the retrospective study, which meant that patients receiving ICIs for longer periods of time had a higher chance of developing documented adverse events. Also, some patients may have ceased using ICIs prior to the development of adverse events. Despite its limitations our study gives valuable insights into the functions of metabolic indicators in predicting the clinical outcome of chemo‐immunotherapy in real‐world scenarios.

## 5 CONCLUSION

Our research suggested that BMI ≥25 kg/m^2^ and elevated levels of cholesterol‐rich apoB‐containing lipoproteins, including LDL‐C and RC, might be attributed to poor therapeutic response to ICIs‐based therapy, particular in advanced NSCLC patients treated with chemotherapy and ICIs combination.

## AUTHOR CONTRIBUTIONS

**Zixin Hu:** Conceptualization (lead); data curation (lead); formal analysis (lead); investigation (lead); methodology (lead); validation (lead); visualization (lead); writing – original draft (lead); writing – review and editing (lead). **Yumin Zheng:** Data curation (equal); formal analysis (supporting); investigation (supporting); validation (lead); writing – review and editing (supporting). **Zhening Liu:** Investigation (supporting); methodology (supporting); validation (supporting); writing – review and editing (supporting). **Jiabin Zheng:** Supervision (lead); writing – review and editing (lead). **Yan Wang:** Data curation (supporting); supervision (equal). **Jiangquan Liao:** Investigation (equal); supervision (supporting). **Jia Li:** Data curation (supporting); investigation (supporting). **Huijuan Cui:** Funding acquisition (lead); methodology (equal); project administration (lead); supervision (lead); writing – review and editing (lead).

## FUNDING INFORMATION

This work was supported by the National High Level Hospital Clinical Research Funding Clinical research (2022‐NHLHCRF‐LX‐02‐0111) & Capital Health Development Scientific Research Project (2022‐2‐4065).

## CONFLICT OF INTEREST STATEMENT

The authors declare that they have no competing interests.

## ETHICS STATEMENT

The study was carried out with the ethical approval of the institutional Ethics Committee of the Faculty of Medicine at China‐Japan Friendship Hospital approval (2022‐KY‐051). All methods were performed in accordance with the relevant guidelines and regulations by including a statement in the declarations (ethics approval and consent to participate) section. Patient consents of ICIs and chemotherapy combination treatment and medication information collection were acquired. Written informed consent was obtained from individual or guardian participants.

## Supporting information


Tables S1–S7.

Figure S1.

Figure S2.

Figure S3.

## Data Availability

The datasets used during the present study are available from the corresponding authors upon reasonable request.
