# Supplementary material for: Effect of body mass index and cholesterol‐rich apolipoprotein‐B‐containing lipoproteins on clinical outcome in NSCLC patients treated with immune checkpoint inhibitors‐based therapy: A retrospective analysis
Source: Cancer Med. 2024 May 31;13(11):e7241. doi: 10.1002/cam4.7241 (PMC11140693; doi:10.1002/cam4.7241)
Supplement: Supplementary file 1 — Tables S1–S7. [file CAM4-13-e7241-s003.docx]

***Table S1 Supplementary clinical features of the 95 patients included in the analysis.***

| Variable | Frequency | N |  |
| --- | --- | --- | --- |
| ICIs medicine |  | 95 |  |
| camr | 1 (1.05%) |  |  |
| durv | 2 (2.11%) |  |  |
| nivo | 5 (5.26%) |  |  |
| nivo+ip | 1 (1.05%) |  |  |
| pemb | 27 (28.4%) |  |  |
| pemb/camr | 1 (1.05%) |  |  |
| sint | 21 (22.1%) |  |  |
| tisl | 35 (36.8%) |  |  |
| tisl/tori | 1 (1.05%) |  |  |
| tori | 1 (1.05%) |  |  |
| Chemotherapy |  | 82 |  |
| A | 4 (4.88%) |  |  |
| AP | 25 (30.5%) |  |  |
| EP | 1 (1.22%) |  |  |
| G | 1 (1.22%) |  |  |
| GP | 2 (2.44%) |  |  |
| nTP | 35 (42.7%) |  |  |
| nT | 12 (14.6%) |  |  |
| T | 1 (1.22%) |  |  |
| TP | 1 (1.22%) |  |  |
| PD-L1(TPS-22C3) |  | 95 |  |
| Not_Available | 68 (71.6%) |  |  |
| <1% | 5 (5.26%) |  |  |
| 1%-49% | 12 (12.6%) |  |  |
| >49% | 10 (10.5%) |  |  |

camr: camrelizumab; durv: durvalumab; nivo: nivolumab; ip: ipilimumab; pemb: pembrolizumab; sint: sintilimab; tisl: tislelizumab; tori: toripalimab; MTA: Pemetrexeddisodium; AP: MTA+Cis-platin/Carboplatin; EP: Etoposide+Cis-platin/Carboplatin; GEM: Gemcitabine; GP: GEM+Cis-platin/Carboplatin; nab-P: Paclitaxel for Injection (Albumin Bound); n-TP: nab-P+Cis-platin/Carboplatin; PTX: Paclitaxel; TP: PTX+Cis-platin/Carboplatin

***Table*** ***S2 Comparison of the clinical features in the groups with BMI < 25 kg/m2 and BMI ≥ 25 kg/m2.***

|  | All  (n=95) | BMI < 25kg /m2  (n=56) | BMI ≥ 25kg /m2  (n=39) | P.val |
| --- | --- | --- | --- | --- |
| Age | 63.1 (9.84) | 63.4 (10.5) | 62.6 (8.98) | 0.722 |
| Gender: |  |  |  | 0.884 |
| Female | 14 (14.7%) | 9 (16.1%) | 5 (12.8%) |  |
| Male | 81 (85.3%) | 47 (83.9%) | 34 (87.2%) |  |
| Stage: |  |  |  | 0.030 |
| IIIa | 9 (9.47%) | 2 (3.57%) | 7 (17.9%) |  |
| IIIb-IV | 86 (90.5%) | 54 (96.4%) | 32 (82.1%) |  |
| Treatment plan: |  |  |  | 1.000 |
| Chemo+ICIs | 82 (86.3%) | 48 (85.7%) | 34 (87.2%) |  |
| ICIs | 13 (13.7%) | 8 (14.3%) | 5 (12.8%) |  |
| Line: |  |  |  | 0.264 |
| 1 | 68 (71.6%) | 43 (76.8%) | 25 (64.1%) |  |
| 2+ | 27 (28.4%) | 13 (23.2%) | 14 (35.9%) |  |
| Smoking history: |  |  |  | 0.738 |
| N | 24 (25.8%) | 13 (23.6%) | 11 (28.9%) |  |
| Y | 69 (74.2%) | 42 (76.4%) | 27 (71.1%) |  |
| Diabetes: |  |  |  | 0.163 |
| N | 76 (81.7%) | 48 (87.3%) | 28 (73.7%) |  |
| Y | 17 (18.3%) | 7 (12.7%) | 10 (26.3%) |  |
| Statin usage: |  |  |  | 1.000 |
| N | 65 (68.4%) | 38 (67.9%) | 27 (69.2%) |  |
| Y | 30 (31.6%) | 18 (32.1%) | 12 (30.8%) |  |
| Histology: |  |  |  | 0.878 |
| LUAD | 55 (57.9%) | 33 (58.9%) | 22 (56.4%) |  |
| LUSC | 33 (34.7%) | 18 (32.1%) | 15 (38.5%) |  |
| NSCLC | 6 (6.32%) | 4 (7.14%) | 2 (5.13%) |  |
| NSCLC+SCLC | 1 (1.05%) | 1 (1.79%) | 0 (0.00%) |  |

LUAD: lung adenocarcinoma; LUSC: lung squamous cell carcinoma; NSCLC: non-small cell lung carcinoma;

***Table S3 Lipoproteins, apolipoproteins, and serum lipids of the BMI < 25 kg/m2 and BMI ≥ 25 kg/m2 groups.***

|  | All  (n=95) | BMI < 25kg /m2  (n=56) | BMI ≥ 25kg /m2  (n=39) | P.val |
| --- | --- | --- | --- | --- |

| Apo A1 | 1.16 (0.26) | 1.16 (0.28) | 1.16 (0.24) | 0.958 |
| --- | --- | --- | --- | --- |
| Apo B | 0.96 (0.28) | 0.98 (0.32) | 0.93 (0.21) | 0.387 |
| Apo E | 3.52 (1.35) | 3.51 (1.47) | 3.54 (1.20) | 0.939 |
| Apo C3 | 8.62 (4.51) | 8.42 (4.48) | 8.85 (4.64) | 0.741 |
| Apo C2 | 3.82 (2.19) | 3.98 (2.56) | 3.64 (1.71) | 0.575 |
| CHO | 4.59 (1.25) | 4.66 (1.32) | 4.48 (1.14) | 0.486 |
| TG | 1.61 (0.85) | 1.50 (0.82) | 1.76 (0.89) | 0.160 |
| HDL-C | 1.09 (0.28) | 1.13 (0.30) | 1.03 (0.23) | 0.060 |
| LDL-C | 2.95 (0.86) | 2.98 (0.95) | 2.89 (0.73) | 0.610 |
| RC | 0.55 (0.29) | 0.55 (0.27) | 0.56 (0.32) | 0.825 |

BMI: body mass index; CHO: cholesterol; TG: ApoB: apolipoproteins B; LDL-C: low-density lipoproteins cholesterol; HDL-C: High-density lipoprotein cholesterol; RC: Remnant cholesterol

***Table S4 Comparison of the clinical features in the groups with DCB and NDB.***

|  | All  (n=74) | DCB  (n=43) | NDB  (n=31) | P.val |
| --- | --- | --- | --- | --- |
| Age | 62.3 (10.7) | 62.6 (11.0) | 61.8 (10.5) | 0.776 |
| Sex: |  |  |  | 0.478 |
| Female | 9 (12.2%) | 4 (9.30%) | 5 (16.1%) |  |
| Male | 65 (87.8%) | 39 (90.7%) | 26 (83.9%) |  |
| Stage: |  |  |  | 0.443 |
| IIIa | 7 (9.46%) | 3 (6.98%) | 4 (12.9%) |  |
| IIIb-IV | 67 (90.5%) | 40 (93.0%) | 27 (87.1%) |  |
| TrPl: |  |  |  | 0.478 |
| Chemo+ICIs | 65 (87.8%) | 39 (90.7%) | 26 (83.9%) |  |
| ICIs | 9 (12.2%) | 4 (9.30%) | 5 (16.1%) |  |
| Line: |  |  |  | 0.508 |
| 1 | 52 (70.3%) | 32 (74.4%) | 20 (64.5%) |  |
| 2+ | 22 (29.7%) | 11 (25.6%) | 11 (35.5%) |  |
| BMI: |  |  |  | 0.007 |
| BMI<25kg/m2 | 41 (55.4%) | 30 (69.8%) | 11 (35.5%) |  |
| BMI≥25kg/m2 | 33 (44.6%) | 13 (30.2%) | 20 (64.5%) |  |
| Smoking_History: |  |  |  | 0.131 |
| N | 18 (25.0%) | 7 (17.1%) | 11 (35.5%) |  |
| Y | 54 (75.0%) | 34 (82.9%) | 20 (64.5%) |  |
| DM: |  |  |  | 0.747 |
| N | 61 (84.7%) | 34 (82.9%) | 27 (87.1%) |  |
| Y | 11 (15.3%) | 7 (17.1%) | 4 (12.9%) |  |
| Statins: |  |  |  | 0.712 |
| N | 52 (70.3%) | 29 (67.4%) | 23 (74.2%) |  |
| Y | 22 (29.7%) | 14 (32.6%) | 8 (25.8%) |  |
| Histology: |  |  |  | 0.148 |
| LUAD | 42 (56.8%) | 24 (55.8%) | 18 (58.1%) |  |
| LUSC | 26 (35.1%) | 13 (30.2%) | 13 (41.9%) |  |
| NSCLC | 5 (6.76%) | 5 (11.6%) | 0 (0.00%) |  |
| NSCLC+SCLC | 1 (1.35%) | 1 (2.33%) | 0 (0.00%) |  |

DCB: durable clinical benefit; NDB = non-durable benefit; LUAD: lung adenocarcinoma; LUSC: lung squamous cell carcinoma; NSCLC: non-small cell lung carcinoma; BMI: body mass index;

***Table S5 Lipoproteins, apolipoproteins, and serum lipids of the DCB and NDB groups.***

|  | All  (n=74) | DCB  (n=43) | NDB  (n=31) | P.val |
| --- | --- | --- | --- | --- |

| Apo-A1 | 1.13 (0.27) | 1.13 (0.26) | 1.13 (0.30) | 0.956 |
| --- | --- | --- | --- | --- |
| Apo-B | 0.97 (0.29) | 0.97 (0.30) | 0.98 (0.29) | 0.909 |
| Apo-E | 3.53 (1.50) | 3.31 (0.90) | 3.83 (2.07) | 0.317 |
| Apo-C3 | 8.47 (5.02) | 7.84 (3.65) | 9.51 (6.74) | 0.405 |
| Apo-C2 | 3.81 (2.31) | 3.99 (2.58) | 3.52 (1.83) | 0.527 |
| CHO | 4.54 (1.24) | 4.38 (1.17) | 4.78 (1.32) | 0.201 |
| TG | 1.63 (0.84) | 1.56 (0.74) | 1.73 (0.98) | 0.425 |
| HDL-C | 1.06 (0.27) | 1.06 (0.28) | 1.07 (0.26) | 0.860 |
| LDL-C | 2.93 (0.86) | 2.83 (0.86) | 3.07 (0.86) | 0.264 |
| RC | 0.55 (0.28) | 0.49 (0.22) | 0.64 (0.34) | 0.042 |

DCB: durable clinical benefit; NDB: non-durable benefit; CHO: cholesterol; TG: ApoB: apolipoproteins B; LDL-C: low-density lipoproteins cholesterol; HDL-C: High-density lipoprotein cholesterol; RC: Remnant cholesterol

***Table S6 Comparison of the clinical characteristics based on the best response (active/non-active) from ICIs.***

|  | All  (n=85) | Active  (n=58) | non_active (n=27) | P.val |
| --- | --- | --- | --- | --- |
| Age | 62.8 (10.2) | 63.0 (10.3) | 62.3 (10.3) | 0.770 |
| Sex: |  |  |  | 0.508 |
| Female | 12 (14.1%) | 7 (12.1%) | 5 (18.5%) |  |
| Male | 73 (85.9%) | 51 (87.9%) | 22 (81.5%) |  |
| Stage: |  |  |  | 0.258 |
| IIIA | 8 (9.41%) | 4 (6.90%) | 4 (14.8%) |  |
| IIIB-IV | 77 (90.6%) | 54 (93.1%) | 23 (85.2%) |  |
| Treatment plan: |  |  |  | 0.135 |
| Chemo+ICIs | 76 (89.4%) | 54 (93.1%) | 22 (81.5%) |  |
| ICIs | 9 (10.6%) | 4 (6.90%) | 5 (18.5%) |  |
| Line: |  |  |  | 0.069 |
| 1 | 60 (70.6%) | 45 (77.6%) | 15 (55.6%) |  |
| 2+ | 25 (29.4%) | 13 (22.4%) | 12 (44.4%) |  |
| BMI: |  |  |  | 0.003 |
| BMI<25kg/m2 | 50 (58.8%) | 41 (70.7%) | 9 (33.3%) |  |
| BMI≥25kg/m2 | 35 (41.2%) | 17 (29.3%) | 18 (66.7%) |  |
| Smoking history: |  |  |  | 0.345 |
| N | 21 (25.0%) | 12 (21.1%) | 9 (33.3%) |  |
| Y | 63 (75.0%) | 45 (78.9%) | 18 (66.7%) |  |
| DM: |  |  |  | 0.510 |
| N | 72 (85.7%) | 50 (87.7%) | 22 (81.5%) |  |
| Y | 12 (14.3%) | 7 (12.3%) | 5 (18.5%) |  |
| Statins: |  |  |  | 1.000 |
| N | 59 (69.4%) | 40 (69.0%) | 19 (70.4%) |  |
| Y | 26 (30.6%) | 18 (31.0%) | 8 (29.6%) |  |
| Histology: |  |  |  | 0.390 |
| LUAD | 50 (58.8%) | 34 (58.6%) | 16 (59.3%) |  |
| LUSC | 29 (34.1%) | 18 (31.0%) | 11 (40.7%) |  |
| NSCLC | 5 (5.88%) | 5 (8.62%) | 0 (0.00%) |  |
| NSCLC+SCLC | 1 (1.18%) | 1 (1.72%) | 0 (0.00%) |  |

ICIs: Immune checkpoint inhibitors; LUAD: lung adenocarcinoma; LUSC: lung squamous cell carcinoma; NSCLC: non-small cell lung carcinoma; BMI: body mass index;

***Table S7 Lipoproteins, apolipoproteins, and serum lipids based on the best response (active/non-active) from ICIs.***

|  | All  (n=85) | Active  (n=58) | non_active (n=27) | P.val |
| --- | --- | --- | --- | --- |
| Apo-A1 | 1.16 (0.27) | 1.15 (0.24) | 1.19 (0.34) | 0.627 |
| Apo-B | 0.96 (0.28) | 0.95 (0.29) | 0.98 (0.27) | 0.641 |
| Apo-E | 3.53 (1.41) | 3.36 (0.92) | 3.97 (2.19) | 0.293 |
| Apo-C3 | 8.62 (4.62) | 8.10 (3.42) | 9.73 (6.52) | 0.393 |
| Apo-C2 | 3.74 (2.19) | 3.83 (2.35) | 3.53 (1.87) | 0.655 |
| CHO | 4.56 (1.21) | 4.44 (1.19) | 4.85 (1.25) | 0.170 |
| TG | 1.62 (0.85) | 1.55 (0.77) | 1.76 (1.02) | 0.378 |
| HDL-C | 1.09 (0.27) | 1.09 (0.27) | 1.11 (0.29) | 0.734 |
| LDL-C | 2.93 (0.84) | 2.84 (0.87) | 3.12 (0.76) | 0.159 |
| RC | 0.54 (0.28) | 0.51 (0.24) | 0.62 (0.35) | 0.146 |

ICIs: Immune checkpoint inhibitors; CHO: cholesterol; TG: ApoB: apolipoproteins B; LDL-C: low-density lipoproteins cholesterol; HDL-C: High-density lipoprotein cholesterol; RC: Remnant cholesterol
