# Supplementary material for: Effect of body mass index and cholesterol‐rich apolipoprotein‐B‐containing lipoproteins on clinical outcome in NSCLC patients treated with immune checkpoint inhibitors‐based therapy: A retrospective analysis
Source: Cancer Med. 2024 May 31;13(11):e7241. doi: 10.1002/cam4.7241 (PMC11140693; doi:10.1002/cam4.7241)
Supplement: Supplementary file 2 — Figure S1. [file CAM4-13-e7241-s002.zip › cam47241-sup-0002-FigureS1 .pdf]

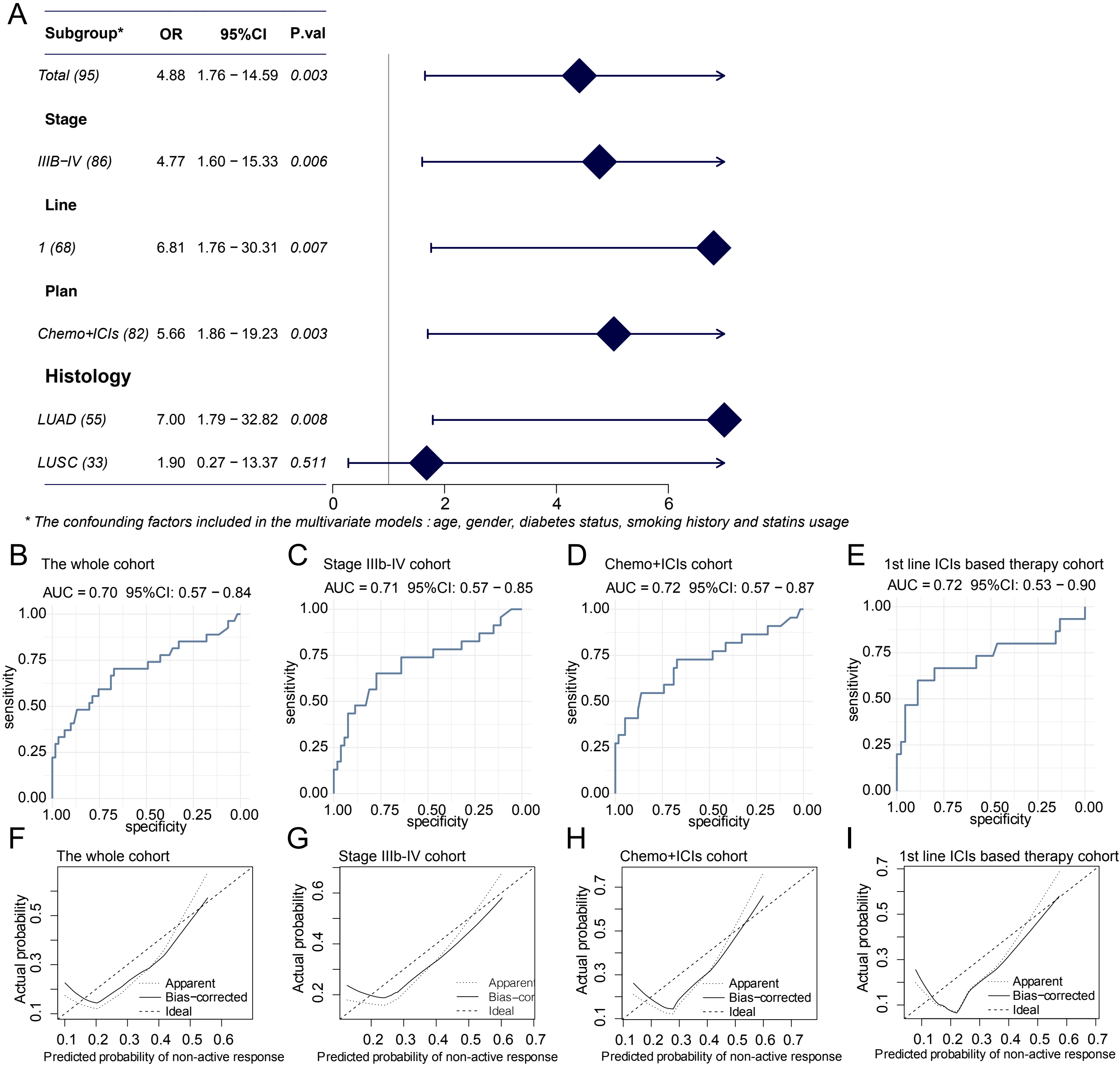
A
Subgroup* OR 95%CI P.val
Total (95) 4.88 1.76 − 14.59 0.003
Stage
IIIB−IV (86) 4.77 1.60 − 15.33 0.006
Line
1 (68) 6.81 1.76 − 30.31 0.007
Plan
Chemo+ICIs (82) 5.66 1.86 − 19.23 0.003
Histology
LUAD (55) 7.00 1.79 − 32.82 0.008
LUSC (33) 1.90 0.27 − 13.37 0.511
0 2 4 6
* The confounding factors included in the multivariate models : age, gender, diabetes status, smoking history and statins usage
B
The whole cohort
AUC = 0.70 95%CI: 0.57 − 0.84
C
Stage IIIb-IV cohort
AUC = 0.71 95%CI: 0.57 − 0.85
D
Chemo+ICIs cohort
AUC = 0.72 95%CI: 0.57 − 0.87
E
1st line ICIs based therapy cohort
AUC = 0.72 95%CI: 0.53 − 0.90
sensitivity
specificity
F
The whole cohort
G
Stage IIIb-IV cohort
H
Chemo+ICIs cohort
I
1st line ICIs based therapy cohort
Actual probability
Predicted probability of non-active response
Apparent
Bias−corrected
Ideal
