# Supplementary material for: Effect of body mass index and cholesterol‐rich apolipoprotein‐B‐containing lipoproteins on clinical outcome in NSCLC patients treated with immune checkpoint inhibitors‐based therapy: A retrospective analysis
Source: Cancer Med. 2024 May 31;13(11):e7241. doi: 10.1002/cam4.7241 (PMC11140693; doi:10.1002/cam4.7241)
Supplement: Supplementary file 2 — Figure S1. [file CAM4-13-e7241-s002.zip › Figure S1 Caption.docx]

Figure S1 Associations between body mass index (BMI) and therapeutic response to immune checkpoint inhibitors (ICIs) based therapy in non-small cell lung cancer patients. (A) Subgroup analysis of the multivariate model to predict non-active response. BMI ≥25 kg/m2 identified as a risk factor for non-active response, with the odds ratio (OR) indicating the increased risk associated with BMI ≥25 kg/m2. (B-E) ROC curves of the multivariate model containing BMI ≥25 kg/m2 in predicting best response to ICIs-based therapy in the whole group and subgroups. (F-I) Calibration curves of the model containing BMI ≥25 kg/m2 in predicting best response in the whole group and subgroups.
