# Supplementary material for: Effect of body mass index and cholesterol‐rich apolipoprotein‐B‐containing lipoproteins on clinical outcome in NSCLC patients treated with immune checkpoint inhibitors‐based therapy: A retrospective analysis
Source: Cancer Med. 2024 May 31;13(11):e7241. doi: 10.1002/cam4.7241 (PMC11140693; doi:10.1002/cam4.7241)
Supplement: Supplementary file 3 — Figure S2. [file CAM4-13-e7241-s001.zip › Figure S2 Caption.docx]

Figure S2 Model diagnosis of multivariate model containing BMI ≥25 kg/m2 used to predict progression-free survival (PFS) in the ICIs-based therapy treated NSCLC patients. (A-D) ROC curves at 3-year of the multivariate Cox model containing BMI ≥25 kg/m2 in predicting PFS in the entire cohort and subgroups. (E-H) Calibration curves of the model in predicting PFS in the whole cohort and subgroups.
