# Supplementary figures and images for: Effect of body mass index and cholesterol‐rich apolipoprotein‐B‐containing lipoproteins on clinical outcome in NSCLC patients treated with immune checkpoint inhibitors‐based therapy: A retrospective analysis
Source: Cancer Med. 2024 May 31;13(11):e7241. doi: 10.1002/cam4.7241 (PMC11140693; doi:10.1002/cam4.7241)

**A**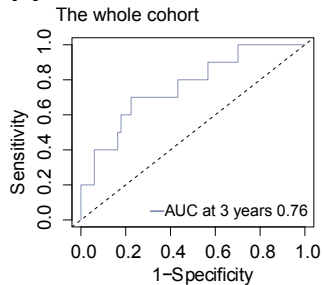**B**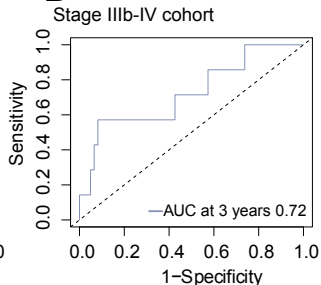**C**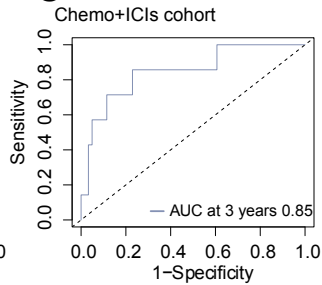**D**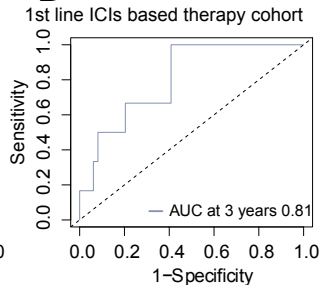**E**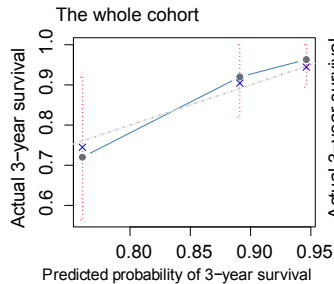**F**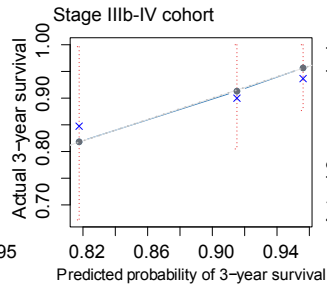**G**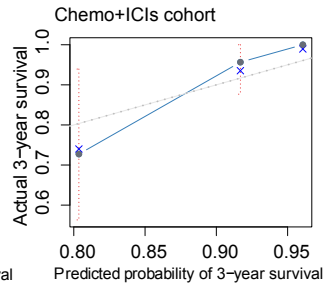**H**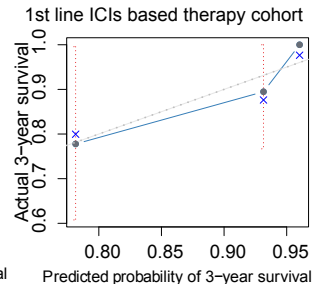

Supplement: Supplementary file 3 — Figure S2. [file CAM4-13-e7241-s001.zip › cam47241-sup-0003-FigureS2 .pdf]

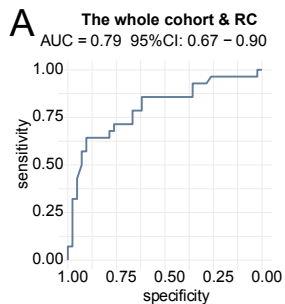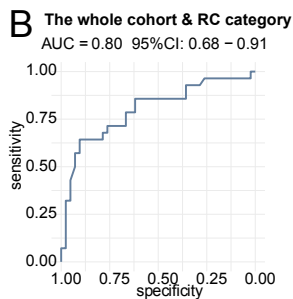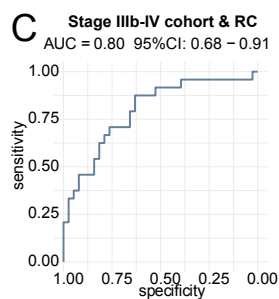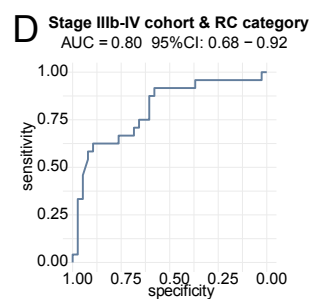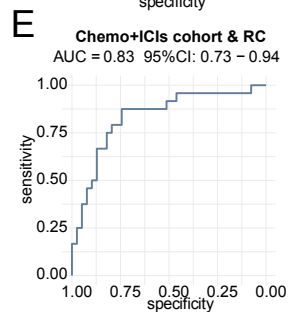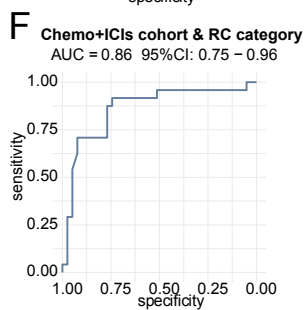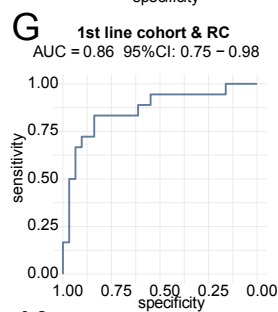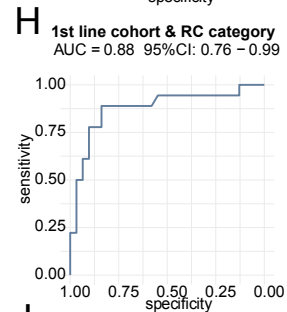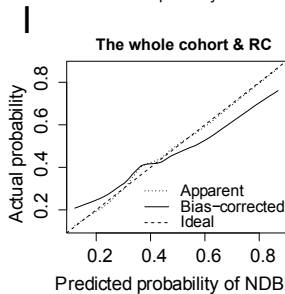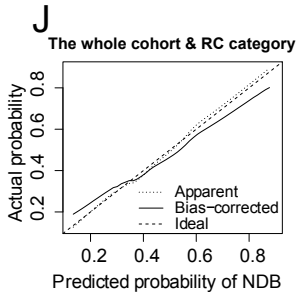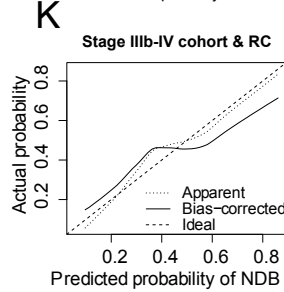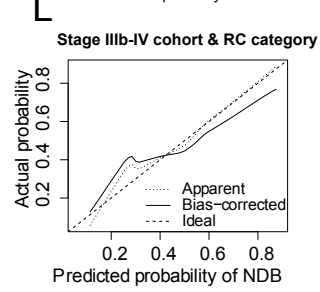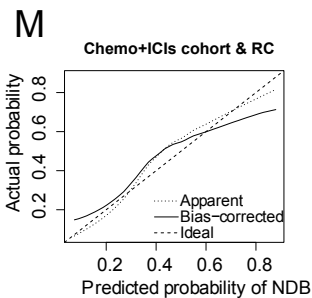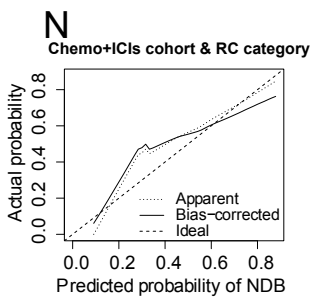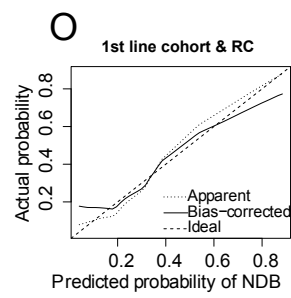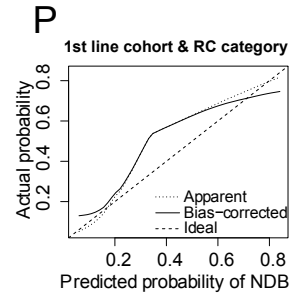

Supplement: Supplementary file 4 — Figure S3. [file CAM4-13-e7241-s004.zip › cam47241-sup-0004-FigureS3 .pdf]
