# Supplementary material for: Effect of body mass index and cholesterol‐rich apolipoprotein‐B‐containing lipoproteins on clinical outcome in NSCLC patients treated with immune checkpoint inhibitors‐based therapy: A retrospective analysis
Source: Cancer Med. 2024 May 31;13(11):e7241. doi: 10.1002/cam4.7241 (PMC11140693; doi:10.1002/cam4.7241)
Supplement: Supplementary file 4 — Figure S3. [file CAM4-13-e7241-s004.zip › Figure S3 Caption.docx]

Figure S3 Model diagnosis of multivariate logistic regression models with remnant cholesterol (RC) in continuous or categorical form for predicting therapeutic response to ICIs (Durable clinical benefit [DCB] vs Non-durable benefit [NDB]) in NSCLC patients treated with ICIs-based therapy. (A-H) ROC curves at 3-year of the multivariate Cox model containing RC in predicting PFS in the entire cohort and subgroups. (I-P) Calibration curves of the model in predicting PFS in the whole cohort and subgroups.
